# Locomotion-induced neural activity independent of auditory feedback in the mouse inferior colliculus

**DOI:** 10.1016/j.isci.2026.115057

**Published:** 2026-02-17

**Authors:** Jisoo Han, Haiyan Jiang, Young Rae Ji, Gunsoo Kim

**Affiliations:** 1 Sensory and Motor Systems Research Group, Korea Brain Research Institute (KBRI), Daegu 41068, South Korea; 2 Center for Neuroscience Imaging Research (CNIR), Institute for Basic Science (IBS), Suwon 16419, South Korea; 3 Department of Biomedical Engineering, Sungkyunkwan University, Suwon 16419, South Korea

**Keywords:** behavioral neuroscience, systems biology

## Abstract

Accumulating evidence indicates that the auditory system integrates movement-related signals with sensory input, yet the mechanisms across processing levels remain incompletely understood. The inferior colliculus (IC), a major midbrain integration center, exhibits locomotion-related neural activity, indicating that midbrain auditory neurons are sensitive to ongoing movement. However, their high sensitivity to sound makes it difficult to distinguish auditory feedback from other motor-related signals. To isolate non-auditory contributions, we recorded IC neural activity in deafened, head-fixed mice walking on a passive treadmill, eliminating auditory feedback through both air and bone conduction. Even in the absence of auditory input, IC neurons showed robust, bidirectional modulation during locomotion. Modulation often began before movement onset, even when aligned to electromyographic rather than treadmill signals, indicating both predictive and feedback components. These results demonstrate that non-auditory, movement-related signals significantly shape neural activity at the midbrain level, potentially supporting rapid, adaptive behavioral responses to sounds during locomotion.

## Highlights

• Non-auditory locomotion-induced IC activity was isolated by eliminating auditory feedback
• IC neurons are bidirectionally modulated during locomotion in the absence of auditory feedback
• Neural modulation can precede movement onset, indicating both predictive and feedback signals

## Graphical abstract

**Figure:**
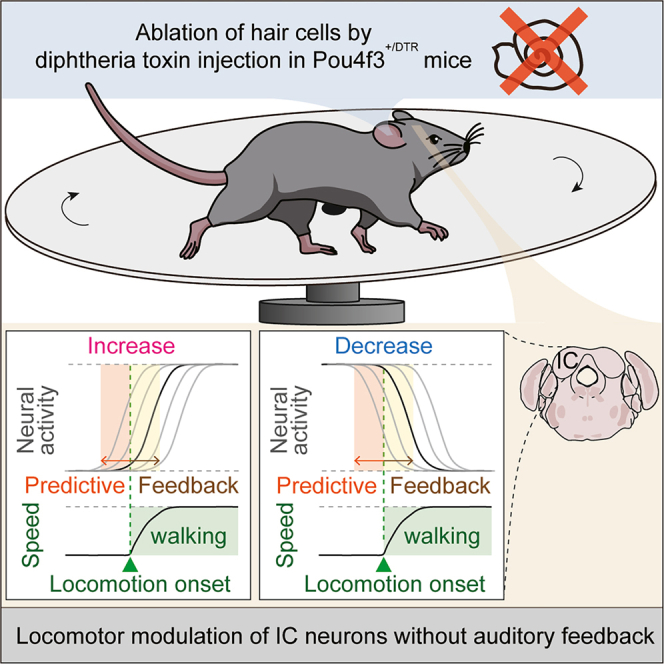

## Introduction

Locomotion is a fundamental behavior across species and strongly influences neural activity of the sensory pathways.^1^^,^^2^^,^^3^^,^^4^ In the auditory system, prior studies have shown that both spontaneous and sound-evoked activity are modulated during locomotion.^5^^,^^6^^,^^7^^,^^8^^,^^9^^,^^10^^,^^11^^,^^12^^,^^13^^,^^14^^,^^15^ During navigation, for example, this modulation may serve important functions, such as encoding movement speed^11^^,^^12^ and distinguishing self-generated sounds from those in the environment.^16^^,^^17^^,^^18^^,^^19^ While locomotion-related modulation has been well characterized in the auditory cortex,^5^^,^^6^^,^^7^^,^^8^^,^^9^^,^^10^^,^^11^^,^^12^ similar modulation has also been observed at subcortical regions, including the auditory thalamus and midbrain.^13^^,^^14^^,^^15^

The inferior colliculus (IC) serves as a major midbrain hub that integrates auditory and other multisensory inputs.^20^^,^^21^ Within the IC, the central nucleus (CNIC) is the primary recipient of the ascending auditory inputs, while the lateral cortex (LCIC) and dorsal cortex (DCIC) also receive multisensory inputs through both ascending and descending projections.^22^^,^^23^^,^^24^^,^^25^^,^^26^^,^^27^^,^^28^^,^^29^^,^^30^ Recent studies have shown that neural activity in the IC is robustly modulated by locomotion,^14^^,^^15^ with two-thirds of the modulated neurons showing increased firing and one-third showing suppression.^15^ These modulations occurred not only in multisensory subregions such as the LCIC and DCIC, but also in the CNIC, traditionally considered a primarily auditory region.^23^^,^^24^^,^^25^^,^^26^^,^^27^ In the DCIC, GABAergic neurons were more strongly correlated with locomotion than glutamatergic neurons, suggesting cell type specific movement-related modulation.^14^ While these findings indicate that auditory neurons in the IC integrate movement-related information, prior studies have not distinguished the relative contributions of auditory feedback and non-auditory, motor-related signals to this modulation.

Locomotion generates sounds that provide auditory feedback, providing information about self-motion and the surrounding environment and potentially influencing ongoing movement.^17^^,^^31^^,^^32^^,^^33^ Moreover, locomotion may induce skull vibrations that stimulate cochlear hair cells via bone conduction,^34^ providing an additional pathway for auditory feedback, but the potential contribution of this mechanism has not been examined. Given the sensitivity of the IC, particularly the CNIC, to acoustic input, such feedback likely contributes to the observed modulation. However, prior studies in hearing mice could not separate the effects of auditory feedback from other motor-related signals, making it difficult to identify the role of non-auditory inputs. While locomotion-related modulation has been reported in hearing-impaired mice,^15^ residual auditory function due to incomplete deafening complicates interpretation. As a result, movement-related neural signals isolated from auditory input have not previously been demonstrated in the IC. Characterizing these non-auditory components in isolation is therefore an important step toward understanding the neural circuits underlying auditory-motor integration in the IC.

In this study, we recorded IC neural activity during locomotion in genetically engineered mice rendered deaf via diphtheria toxin (DT)-induced ablation of cochlear hair cells (Pou4f3^+/DTR^ line),^35^ effectively eliminating auditory feedback through both air and bone conduction. We observed clear modulation of IC activity during locomotion, even in the absence of auditory input. Temporal analysis of neural modulation relative to locomotion onset indicated the presence of both predictive and feedback components. Our findings demonstrate that non-auditory, movement-related signals significantly shape neural activity in the auditory midbrain, likely contributing to auditory-motor integration during behavior.

## Result

### Locomotion-induced auditory feedback in head-fixed mice

**Figure 1:**
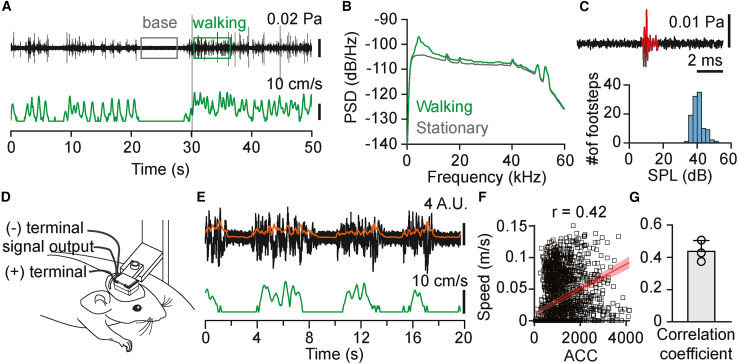
Locomotion generates detectable auditory feedback in head-fixed mice (A) Top, example sound pressure waveform recorded near the forepaw during locomotion (black trace; bandpass filtered between 1 and 50 kHz). Boxes indicate stationary (gray) and walking (green) periods used for spectral analysis in (B). Bottom, corresponding walking speed (green trace). (B) Power spectral density of sound recordings during stationary (gray) and walking (green) periods. (C) Example waveform of a single footstep event (top) and histogram of root-mean-square (RMS) sound pressure levels (SPLs) of individual footsteps (bottom). (D) Schematic diagram of the skull-mounted sensor setup for detecting bone-conducted vibrations during walking in head-fixed mice. (E) Example traces showing the raw vibration sensor signal (black), smoothed envelope of the signal (orange; see method details), and walking speed (green). (F) Relationship between walking speed (green trace in E) and the smoothed vibration signal (orange trace in E). Each point represents paired values averaged in 100 ms bins. Waking speed was positively correlated with the smoothed vibration signal (r = 0.42). Solid line, linear regression; shaded area, 95% confidence interval. (G) Correlation between the smoothed vibration signal and walking speed. Bar graph shows the mean correlation coefficient ±SD (r = 0.44 ± 0.07; *n* = 3 mice).

We first investigated whether locomotion in head-fixed mice on a passive treadmill generates significant auditory feedback. To assess air-conducted auditory feedback, we recorded locomotion-generated sounds using a microphone positioned near the mouse’s ear. Soft footstep sounds were detected during locomotion bouts, with most of the sound energy concentrated between 2 and 10 kHz (Figures 1A and 1B). The average sound intensity of individual footsteps was 40 ± 3 dB SPL (mean ± SD; Figure 1C; 109 footsteps, *n* = 1 mouse; range, 35–51 dB). To assess whether locomotion induces significant vibrations under head-fixed conditions, we also measured skull vibrations using a vibration sensor attached to the skull (Figure 1D).^36^ Clear vibration signals were detected during walking bouts and were strongly correlated with walking speed (Figures 1E–1G). These results demonstrate that, even under head-fixed conditions, locomotion generates substantial auditory feedback through both airborne and bone-conducted pathways, likely contributing to neural modulation in the auditory pathway during movement.

### Locomotion modulates IC neural activity in the absence of auditory feedback

**Figure 2:**
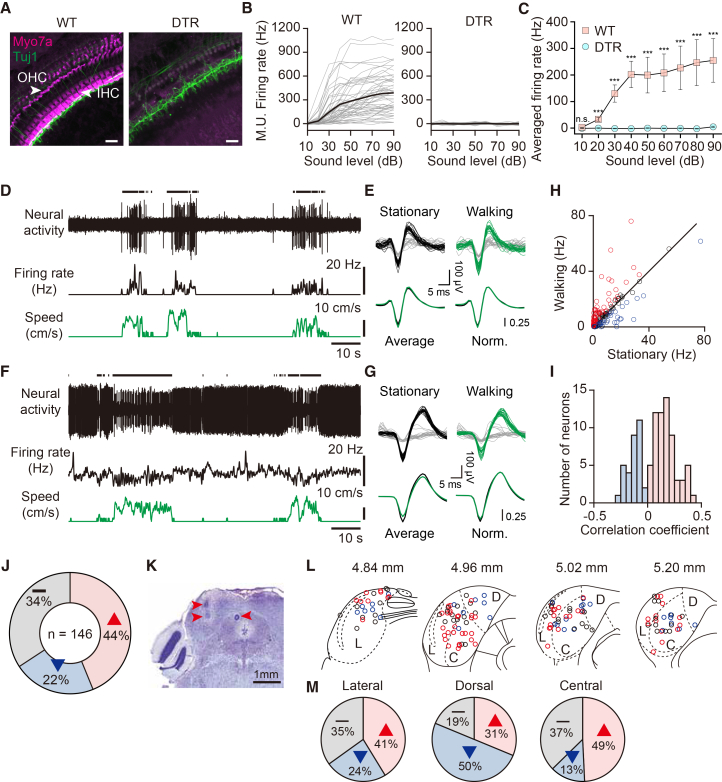
Modulation of IC neural activity during locomotion in deaf mice (A) Representative images of the cochlear apical turn from a WT mouse treated with DT (left) and the middle turn from a DTR mouse (right). In DTR mice injected with diphtheria toxin, hair cells (labeled with myosin VIIa, magenta) are absent, confirming successful ablation. Afferent auditory nerve fibers (Tuj1, green) are also labeled. See also Figure S1. Scale bars: 20 μm. (B) Sound-evoked IC multi-unit firing rates across sound levels (dB) in WT (left) and DTR (right) mice. Each gray trace represents responses from a multi-unit recording site; thick black line indicates the average for the mouse. See also Figure S2. (C) Average sound-evoked firing rates across sound intensities (10–90 dB SPL) in WT (*n* = 3 mice) and DTR mice (*n* = 24 mice) (∗∗∗*p* < 0.001, n.s., not significant, Mann-Whitney U test). Data are shown in mean ± SEM across animals. (D and F) Example IC single-unit recordings in deaf mice exhibiting increased (D) or decreased (E) firing rates during locomotion. Top, raw spike trace; middle, smoothed firing rate; bottom, walking speed. Black bars (above top trace) indicate walking periods. (E and G) Spike waveforms recorded during walking (green) and stationary (black) periods from neurons shown in (D) and (F), respectively (25 randomly selected waveforms per condition; waveforms were collected from 200 s recordings that included the segments shown in [D] and [F]). Spike waveforms not included in the single unit clusters are shown in gray. Normalized average waveforms are highly similar between the two conditions, with correlation coefficients of r = 0.998 (E), r = 0.997 (G). (H) Scatterplot of mean firing rates during stationary vs. walking periods for all IC neurons recorded in deaf mice (*n* = 146 neurons). Red, increased firing; blue, decreased firing; black, not statistically significant changes. Diagonal line indicates unity. (I) Distribution of correlation coefficients between firing rate and walking speed for modulated neurons. Pink bars, increased firing; blue bars, decreased firing. (J) Proportions of neurons in deaf mice with increased (pink), decreased (blue), or unchanged (gray) activity during locomotion. (K) Nissl-stained coronal section showing lesion sites (red arrowheads) used to verify IC recording locations. Only recording sites within the boundaries of the IC were included in the analysis. Scale bars: 1 mm. (L) Spatial distribution of IC neurons recorded in deaf mice across four coronal sections (4.84, 4.96, 5.02, 5.20 mm posterior to bregma). L, lateral cortex; D, dorsal cortex; C, central nucleus. Red, increased firing; blue, decreased firing; black, unmodulated neurons. (M) Proportion of modulation types within each subdivision. Pink, increased firing; blue, decreased firing; gray, not statistically significant changes.

To eliminate auditory feedback during locomotion and isolate non-auditory components of neural modulation in the auditory pathway, we used Pou4f3^+/DTR^ mice, which express the diphtheria toxin receptor (DTR) in cochlear hair cells.^35^ One week after DT injection (25 ng/kg), we assessed deafness using whole-mount cochlear histology and neural recordings. Myosin VIIa immunostaining revealed a near-complete loss of hair cells throughout all cochlear turns in DTR mice (Figure 2A; quantification in Figure S1). Consistent with the anatomical result, multi-unit recordings from the IC showed reliable sound-evoked responses in wildtype (WT) mice (*n* = 3 mice), whereas virtually no detectable sound-evoked responses were observed in DTR mice (*n* = 24 mice), with the exception of one mouse that showed responses only at 90 dB (Figures 2B, 2C, and S2). Elimination of cochlear hair cells should also prevent locomotion-induced skull vibrations from being transduced into neural signals via bone conduction.^34^^,^^37^ Together, these results confirm that DT injection in Pou4f3^+/DTR^ mice effectively induces deafness (hereafter referred to as “deaf mice”), eliminating auditory feedback during locomotion.

To investigate whether IC neural activity is modulated by locomotion in the absence of auditory feedback, we performed single-unit recordings in head-fixed, deaf mice on a passive treadmill. Deafening significantly decreased spontaneous firing rates during stationary periods (hearing: 11.9 ± 1.4 Hz, *n* = 73; deaf: 8.8 ± 0.9 Hz, *n* = 146; mean ± SEM; *p* = 0.0225, U = 4,322, n1 = 73, n2 = 146, Mann-Whitney U test), yet locomotion still induced robust modulation in IC activity (Figure S2C). In an example IC neuron, the average firing rate increased from 0.2 Hz at rest to 3.8 Hz during locomotion (Figure 2D; *p* = 1.04e-17, t = 9.89, df = 136, Student’s *t* test), while another neuron showed a decrease from 10.5 Hz to 8.0 Hz (Figure 2F; *p* = 2.6e-10, t = −6.52, df = 333, Student’s *t* test). Among the 146 IC neurons recorded, 64 neurons (44%) increased their firing during locomotion, exhibiting positive correlations with walking speed, whereas 32 neurons (22%) decreased their firing, exhibiting negative correlations with speed (Figures 2H, 2I, and 2J; Student’s *t* test for each neuron, *p* < 0.01). The remaining 34% of neurons did not show statistically significant change in firing rate during locomotion (Student’s *t* test, *p* > 0.01). These results demonstrate that IC neural activity is robustly modulated by locomotion, even in the absence of auditory feedback, and suggest that the majority of this modulation originates from non-auditory sources.

The shell regions of the IC (LCIC and DCIC) are known to be multisensory, receiving somatosensory inputs, for example, in addition to the auditory inputs.^26^^,^^27^ Therefore, one might expect that non-auditory components of locomotion-induced activity modulation occur primarily in the shell regions. We investigated the spatial distribution of neurons modulated by locomotion across the IC by dividing it into three subdivisions in deaf mice: lateral (L), dorsal (D), and central (C) (Figure 2L).^38^ Our reconstruction of recording locations based on lesions (Figure 2K) revealed that locomotion-induced modulation is observed in all IC subdivisions in deaf mice. We examined the proportions of modulated neurons and their modulation directions in each subregion. In the LCIC and CNIC, excitatory modulation was more prevalent, whereas suppressive modulation was more prominent in the DCIC. Across all subdivisions, including the CNIC, more than 60% of the neurons exhibited sensitivity to locomotion (Figure 2M). These findings demonstrate that locomotion-induced activity modulation occurs across all IC subdivisions, even in deafness, with regional differences in prevalence of the direction of modulation.

### Temporal relationship between IC neural activity modulation and locomotion onset

Neural modulation that precedes movement onset is consistent with a predictive signal such as corollary discharge,^5^ whereas modulation that follows movement onset is more consistent with a feedback signal such as somatosensory inputs induced by movement.^39^^,^^40^ Previous studies have shown that in many IC neurons, neural modulation precedes locomotion onset.^14^^,^^15^ Here, we asked whether the absence of auditory feedback in deaf mice influences the timing of modulation.

**Figure 3:**
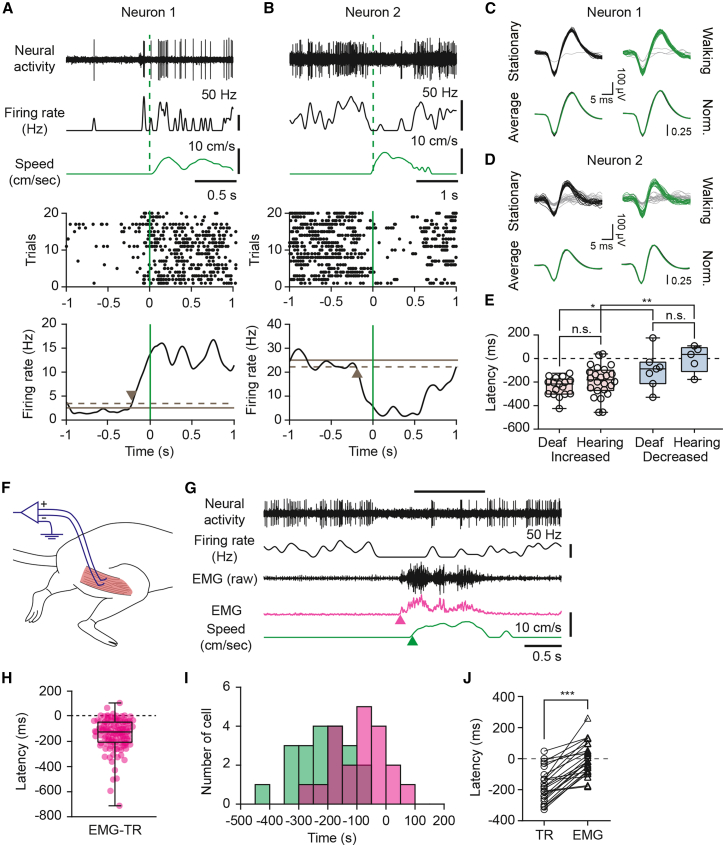
Modulation latency of locomotion-related IC activity in deaf mice (A and B) Examples of IC neurons showing increased (A) or decreased (B) firing at locomotion onset in deaf mice. Top, raw neural activity (top trace), smoothed firing rate (middle trace), and walking speed (bottom trace, green). Green dashed vertical line marks locomotion onset. Middle, raster plots of spike times aligned to locomotion onset across bouts, with green vertical line marking the locomotion onset. Bottom, locomotion onset-triggered average firing rates (black traces). Horizontal solid and dashed lines indicate the mean ±1 SD of baseline activity. Arrowheads indicate the latency of modulation, defined as the time at which the average firing rate first crosses the 1 SD threshold. (C and D) Spike waveforms recorded during walking (green) and stationary (black) periods from the neurons shown in (A) and (B), respectively (25 randomly selected waveforms per condition). Spike waveforms not included in the single unit clusters are shown in gray. Averaged and normalized waveforms overlaid for comparison. Waveform similarity between conditions was high (correlation coefficients: r = 0.99 [C] and r = 0.99 [D]). (E) Comparison of modulation latencies between deaf and hearing mice for neurons with increased firing rates (left; deaf, *n* = 17 neurons; hearing, *n* = 24 neurons) and decreased firing rates (right; deaf: *n* = 7 neurons; hearing: *n* = 5 neurons) activity. n.s., not significant (increased: *p* = 0.14, U = 147.5, n1 = 17, n2 = 24, decreased: *p* = 0.27, U = 10, n1 = 7, n2 = 5, Mann-Whitney U test). Latency comparison between increased and decreased neurons within deaf mice and hearing mice (deaf: ∗*p* = 0.023, U = 24, n1 = 17, n2 = 7, hearing: ∗∗*p* = 0.0069, U = 15, n1 = 24, n2 = 5, Mann-Whitney U test). Box and whiskers plot shows median, interquartile range, and minimum/maximum values. See also Figure S3. (F) Schematic of EMG recording for the hindlimb flexor muscle activity. (G) Simultaneous recording of IC activity, EMG, and treadmill speed during a walking bout. From top to bottom: raw neural activity, smoothed firing rate, raw and smoothed EMG signal (magenta), and walking speed (green). (H) Delays between hindlimb EMG onset and detectable treadmill movement across individual locomotion onsets (*n* = 121 onsets from eight mice). Negative values indicate that EMG onset precedes treadmill movement. Box and whiskers plot shows median, interquartile range, and minimum/maximum values. (I) Distribution of modulation latencies for individual IC neurons in deaf mice. Treadmill-based latencies from (E) are shown in green, and latencies adjusted by the median EMG-treadmill delay from (H) are shown in magenta. (J) Direct comparison of modulation latencies relative to treadmill onset (TR) and EMG onset (EMG) for neurons recorded with simultaneous EMG measurements (*n* = 24 neurons). ∗∗∗*p* = 1.2e-8, t = 8.612, df = 23, paired *t* test.

We determined modulation onset times for each neuron by averaging firing rates across individual walking bouts, as shown in the example neurons (Figures 3A and 3B). In many neurons with increased firing, modulation began before locomotion onset, consistent with previous observations in hearing mice. The median modulation latency did not differ significantly between deaf and hearing mice for either neurons with increased firing (Figure 3E; median latency, −216 ms [deaf] vs. −181 ms [hearing], *p* = 0.14, U = 147.5, n1 = 17, n2 = 24, Mann-Whitney U test) or neurons with decreased firing during locomotion (Figure 3E; median latency, −88 ms [deaf] vs. −35 ms [hearing], *p* = 0.27, U = 10, n1 = 7, n2 = 5, Mann-Whitney U test). Within deaf mice, modulation latency was significantly more negative in excited neurons than in suppressed neurons (Figure 3E; −216 ms vs. −88 ms, *p* = 0.023, U = 24, n1 = 17, n2 = 7, Mann-Whitney U test), similar to hearing mice (−181 ms vs. −35 ms, *p* = 0.0069, U = 15, n1 = 24, n2 = 5, Mann-Whitney U test). This difference may reflect distinct circuit mechanisms underlying suppressive modulation, such as recruitment of feedforward inhibition. No clear spatial pattern of modulation latency was observed across IC subregions (Figure S3).

Because there is a delay between limb muscle activation and the actual movement onset of treadmill movement, some modulation signals that appear to precede locomotion onset may in fact follow muscle activation. To obtain more accurate timing estimates, we recorded electromyographic (EMG) signals from hindlimb flexor muscles in a subset of animals (Figure 3F; see method details).

As illustrated by an example suppressed neuron, hindlimb EMG onset preceded treadmill movement onset (Figure 3G). Across locomotion onsets, the delay between EMG onset and treadmill onset was variable, likely reflecting differences in how walking is initiated across bouts, with a median delay of −125 ms (Figure 3H; *n* = 121 onsets from eight mice). When modulation latencies were adjusted for this median EMG-treadmill delay, the median latency shifted significantly toward more positive values (from −205 ms relative to treadmill onset to −80 ms relative to EMG onset; *p* < 0.001, t = 8.1, df = 21, paired *t* test; Figure 3I).

In a subset of IC recordings, both treadmill and EMG signals were obtained, allowing direct comparison of treadmill- and EMG-based modulation latencies within the same neurons (*n* = 24 neurons, Figure 3J). In these neurons, the median modulation latency also shifted significantly toward more positive values (from −181 ms relative to treadmill onset to −17 ms relative to EMG onset; *p* = 1.2e-8, t = 8.6, df = 23, paired *t* test).

Taken together, these indirect and direct comparisons suggest that treadmill-based measurements tend to overestimate the magnitude and prevalence of pre-movement modulation. Consequently, a larger proportion of the observed activity is likely attributable to somatosensory feedback than previously suggested. Nonetheless, even when referenced to EMG-based onsets, a subpopulation of IC neurons maintained negative latencies, providing evidence for anticipatory, pre-movement signals.

**Figure 4:**
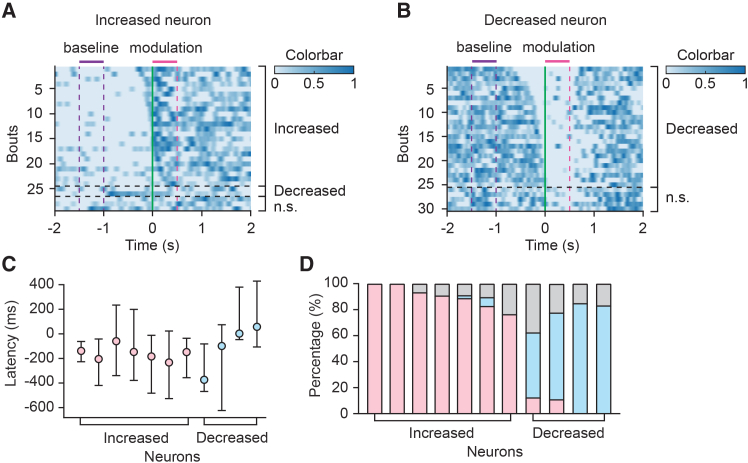
Variability of IC neural modulation across locomotion bouts (A and B) Heatmaps of normalized firing rates from individual locomotion bouts for example IC neurons showing overall increased (A) or decreased (B) firing rates during walking. Each row represents a single locomotion bout, aligned to locomotion onset (green vertical line). Bouts are sorted and grouped by modulation type (increased, decreased, or n.s., not statistically significant), with black horizontal dashed lines indicating group boundaries. Time windows used to measure baseline and modulation around locomotion onset are indicated by horizontal bars and vertical dashed lines. (C) Mean modulation latency for each neuron with overall increased (left, *n* = 7) or decreased (right, *n* = 4) firing during walking. Error bars indicate the range of latencies across bouts. (D) Proportion of bout-wise modulation types for each neuron. Stacked bars show the proportion of locomotion bouts classified as increased (pink), decreased (blue), or not statistically significant (gray) relative to locomotion onset.

To better understand the dynamics of modulatory signals beyond average latency values and overall direction, we examined neural activity around individual locomotion onset events. In neurons where bout-wise analysis was feasible (see method details), modulation onset times varied widely and often straddled the onset of movement (median range, 481 ms for increased neurons [*n* = 7]; 492 ms for decreased neurons [*n* = 4]) (Figures 4A–4C). In contrast, the direction of modulation was highly consistent with each neuron’s average change; modulation in the opposite direction occurred in only ∼3% of bouts across the 11 neurons examined (Figures 4A, 4B, and 4D). This variability in modulation latency likely reflects differences in how individual movements are initiated.

## Discussion

To understand the mechanisms underlying locomotion-induced neural modulation in the IC, it is crucial to isolate the contributions of distinct input sources. In this study, using a mouse line that can be rendered deaf, we were able to separate non-auditory, movement-related neural signals from both air- and bone-conducted auditory feedback. We found that locomotion-related modulation persists even in the absence of auditory input, with both excitatory and suppressive changes observed across all IC subdivisions. Temporal analysis showed that neural modulation often precedes movement onset in deaf mice, similar to hearing animals. In addition, by measuring modulation latency relative to hindlimb EMG signals rather than treadmill motion, we more precisely demonstrate that non-auditory modulation includes both predictive and feedback components. The widespread distribution of this modulation, including in the CNIC, suggests that integrating non-auditory, movement-related information with auditory input is a fundamental feature of IC function.

### Isolation of non-auditory neural modulation in the IC

Our results in deaf mice provide clear evidence that IC neurons exhibit robust non-auditory modulation during locomotion. Compared to prior reports in hearing mice,^15^ the proportion of excitatory modulation decreased from 53% to 44% in deaf mice (Figure 2H), suggesting that approximately 17% of excitation can be attributed to auditory feedback—an effect not separable in previous studies using hearing mice.^14^^,^^15^ In contrast, the proportion of neurons exhibiting suppressive modulation remained unchanged in deaf mice (∼22%), suggesting that the suppression likely is driven by non-auditory inputs. Moreover, the persistence of modulation in the CNIC of deaf animals suggests that CNIC neurons also integrate multi-modal information, which was difficult to demonstrate in hearing mice due to their high auditory sensitivity. The contribution of air-conducted auditory feedback may vary with environmental conditions, and it would be important to investigate how non-auditory signals interact with auditory feedback under different behavioral and sensory contexts.^31^^,^^33^

If locomotion generates skull vibrations, these could propagate through the auditory pathway via bone conduction. Indeed, we detected consistent movement-related skull vibrations even under head-fixed conditions, revealing a previously underappreciated source of auditory feedback. In deaf mice, this bone-conducted component is also eliminated, allowing isolation of non-auditory modulation. Although the precise contribution of bone conduction remains difficult to quantify, it is important to recognize that such signals would persist in the presence of masking noise—an approach commonly used to investigate movement-related, non-auditory neural activity.^5^^,^^14^^,^^41^

Our recordings were performed approximately one week after deafening, raising the question of whether neural plasticity could influence the results. While adult-onset hearing loss could induce plastic changes in auditory and multisensory brain regions,^42^^,^^43^ these changes tend to involve the reorganization of existing circuits rather than the formation of entirely new connections.^42^^,^^43^^,^^44^ Furthermore, recent work has shown that motor-related signals in the auditory cortex are preserved in both congenitally deaf and hearing mice, suggesting that such signals are not highly dependent on auditory experience.^45^ While we cannot completely rule out plastic changes in movement-related IC inputs following deafening, these are likely to be quantitative rather than qualitative.

### Temporal relationship between IC modulation and movement onset

The timing of neural modulation relative to locomotion onset provides insight into whether movement-related signals are predictive or feedback-driven. In most analyses, movement onset was defined by the onset of treadmill motion (Figures 3A, 3B, 3E, and 4). However, since limb muscle activation (as measured by EMG) typically precedes detectable treadmill motion (Figure 3H), somatosensory feedback related to muscle activity may occur before detectable movement, potentially leading to an overestimation of predictive signals.

When modulation latencies were adjusted for the delay between EMG onset to treadmill movement, latencies shifted toward more positive values, and a larger fraction of responses became consistent with feedback signals (Figure 3I). Similarly, in neurons where we simultaneously measured IC neural activity with EMG signals from a hindlimb flexor muscle, modulation latencies shifted toward more positive values when aligned to EMG onset (Figure 3J). These results suggest that a substantial portion of locomotion-related modulation reflects feedback signals, likely mediated by somatosensory pathways.^30^^,^^39^^,^^40^^,^^46^^,^^47^ Despite the shift, a subset of IC neurons continued to exhibit modulation that preceded EMG-based movement onset, indicating the presence of anticipatory signals in at least some neurons. While the neural sources of these anticipatory modulations in the IC remain unclear, potential contributors include midbrain locomotion circuits,^48^^,^^49^ descending projections from motor cortex,^28^^,^^29^ and neuromodulatory systems.^50^^,^^51^

In a small subset of IC neurons, we measured modulation latency at individual locomotion onsets. In these neurons, modulation latency varied considerably across locomotion onsets, even within individual neurons (Figure 4). This variability likely reflects differences in movement initiation patterns, as suggested by the variability in EMG onset times relative to treadmill onset (Figure 3F). Although our dataset does not allow us to correlate specific movement parameters with neural modulation at each bout, the observed variability in IC modulation patterns suggests that locomotion related signals may be sensitive to the dynamics of limb and body movements,^40^ rather than simply reflecting global behavioral states such as arousal.^7^^,^^52^ Instances of modulation in the direction opposite to a neuron’s dominant response, such as transient suppression in an otherwise excited neuron, were rare (Figure 4D). While bout-wise analysis was limited to only in a small number of neurons, this finding contrasts with reports of variable modulation polarity in auditory cortical neurons during vocalization,^45^ potentially reflecting differences in modulatory circuit organization between cortical and subcortical auditory regions, differences in motor behaviors, or both.

While analyses based on timing relative to movement onset are informative, they are inherently limited in that once locomotion begins, predictive and feedback signals overlap temporally, making them difficult to disentangle. Dissecting these components will require targeted manipulation of specific input pathways. Our deafened mouse model, which excludes auditory feedback, provides a valuable framework for such investigations by isolating non-auditory locomotion-related inputs.

### Movement and the auditory pathway

Our findings raise broader questions about how movement-related information is integrated across different levels of the auditory pathway to support behavior. It is well established that auditory cortical neurons are modulated by locomotion and other motor behaviors.^4^ While the sources of modulation may differ depending on specific behaviors, growing evidence points to the involvement of direct non-auditory inputs to the auditory cortex, rather than inheritance from the auditory thalamus.^5^^,^^41^^,^^50^^,^^53^ On the other hand, recent evidence including our current results indicate that movement-related modulation is already prominent in subcortical auditory regions, including the cochlear nucleus, IC, and auditory thalamus.^13^^,^^14^^,^^15^^,^^54^ This raises questions about how modulation is distributed and transformed along the auditory hierarchy.

Different stages of the auditory pathway may encode movement-related signals via distinct mechanisms. For example, the IC receives direct ascending and descending somatosensory inputs, which are likely engaged during locomotion.^40^^,^^47^ In addition, while the IC exhibits modulation in both spontaneous and evoked firing, in the medial geniculate body (MGB), locomotion primarily attenuates sound-evoked responses without significantly affecting spontaneous activity.^7^^,^^13^ Although the downstream impact of IC modulation on MGB activity remains to be fully elucidated, long-range GABAergic neurons in the IC shell that receive cortical somatosensory inputs have been shown to suppress auditory responses in the thalamus.^46^ Similar non-auditory modulation of these GABAergic projection neurons during movement could therefore contribute to the suppression of MGB activity. Together, these anatomical and physiological differences across subcortical and cortical auditory regions raise the possibility that locomotion-related modulations arise from different mechanisms and may even serve distinct functions at successive stages of auditory processing.

The functional significance of movement-induced modulation in the auditory pathway remains an active area of investigation. Recent studies suggest that auditory cortical populations encode behaviorally relevant variables such as running speed and prediction error, indicating they play a role in monitoring self-generated movement and its sensory consequences.^11^^,^^12^^,^^18^^,^^19^^,^^31^^,^^55^ Our observation of significant correlations between firing rate and locomotion speed at the single neuron level in the IC (Figure 2G) suggests that elements of this encoding already emerge at the midbrain level. This aligns with growing evidence that IC neurons are sensitive to a wide array of behavioral and cognitive parameters.^56^^,^^57^^,^^58^^,^^59^ Tracking movement-related information at early stages of auditory processing may enable rapid, context-dependent responses to environmental sounds, supporting adaptive behavior during active exploration.

### Limitations of the study

Although we established the effectiveness of deafening, cochlear histology was not performed in every animal used for recordings. To address this limitation, we also mapped sound-evoked activity in the IC during recordings. While these recordings revealed no detectable sound-evoked responses at nearly all sites, it remains possible that residual responses were present but not detected by our mapping. In addition, we infer the elimination of bone-conducted auditory responses based on histological evidence and the absence of air-conducted responses, rather than on direct measurements of bone-conducted signals.

## Resource availability

### Lead contact

Requests for further information and resources should be directed to and will be fulfilled by the lead contact, Gunsoo Kim (kgunsoo@kbri.re.kr).

### Materials availability

This study did not generate new unique reagents.

### Data and code availability

• Data reported in this article will be shared by the lead contact upon request.
• This study does not report original code.
• Any additional information required to reanalyze the data reported in this article is available from the lead contact upon request.

## Acknowledgments

This study was supported by NRF-2021R1F1A1049434 to G.K. and Brain Pool program (NRF-2022H1D3A2A02062811) to G.K. by the Ministry of Science and ICT through the National Research Foundation of Korea, KBRI basic research program (26-BR-01-01 and 26-BR-07-01) to G.K. by the Ministry of Science and ICT through Korea Brain Research Institute, and 10.13039/501100010446Institute for Basic Science (IBS-R015-D1). We thank Siyoon Oh for her technical assistance with mouse genotyping and Dr. Mimi Kao for her critical comments on earlier versions of the manuscript.

## Author contributions

Conceptualization, J.H. and G.K.; methodology, J.H., H.J., and G.K.; investigation, J.H., H.J., Y.R.J., and G.K.; writing – original draft, J.H. and G.K.; writing – review and editing, J.H., H.J., and G.K.; funding acquisition, G.K.

## Declaration of interests

The authors declare no competing interests.

## STAR★Methods

### Key resources table

**Table:** 

REAGENT or RESOURCE	SOURCE	IDENTIFIER
**Antibodies**		

Rabbit polyclonal anti-Myosin VIIa	Proteus Biosciences	Cat#25–6790; RRID: AB_10015251
Mouse Monoclonal anti-beta III Tubulin (Tuj1)	Biotechne R&D systems	Cat#MAB1195; RRID: AB_357520
Goat anti-Rabbit IgG (H + L) Cross-Adsorbed Secondary Antibody, Alexa Fluor™ 405	Thermo	A-31556; RRID: AB_221605
Goat anti-Mouse IgG2a Cross-Adsorbed Secondary Antibody, Alexa Fluor™ 488	Thermo	A-21131; RRID: AB_2535771

**Chemicals, peptides, and recombinant proteins**		

Diphtheria Toxin and CRM	List Labs	Cat#150
Alexa Fluor 647 phalloidin	Thermo	A22287

**Experimental models: Organisms/strains**		

Mouse:C57BL/6J	Jackson Laboratory	JAX#000664
Mouse: B6.Cg-Pou4f3tm1.1(HBEGF)Jsto/RubelJ	Jackson Laboratory	JAX#028673

**Software and algorithms**		

ImageJ	NIH	https://imagej.net/software/imagej/
Open Ephys GUI (v.0.5.5)	Open Ephys	https://open-ephys.org/
MATLAB (R2020b)	Mathworks	https://mathworks.com

### Experimental model and study participant details

#### Animals

All procedures were approved by the Institutional Animal Care and Use Committee (IACUC-23-00077-M2) of the Korea Brain Research Institute and complied with institutional guidelines. Mice were group-housed under a 12:12-h light/dark cycle with *ad libitum* access to food and water. Animal housing, care, and routine health monitoring were provided by the institute’s animal care facility. Experiments were conducted using C57BL/6J mice (*N* = 19) and Pou4f3^+/DTR^ mice (*N* = 35) of both sexes, aged 6–15 weeks. Deafness was induced in Pou4f3^+/DTR^ mice via a single intramuscular injection of diphtheria toxin (25 ng/kg) at least 7 days prior to neural recording.

### Method details

#### Headpost surgery

Before surgery, the stereotaxic apparatus and surgery area was disinfected, and all surgical instruments were sterilized. Mice were anesthetized via intraperitoneal injection of ketamine (100 mg/kg, Yuhan) and xylazine (10 mg/kg, Bayer Korea). Depth of anesthesia was assessed using a tail or tow pinch, and body temperature was maintained at 37° with a heating pad. Eye ointment was applied to prevent corneal drying. After shaving the scalp, the head was secured in a stereotaxic apparatus, and lidocaine was injected subcutaneously for local anesthesia. The scalp was disinfected with povidone-iodine, a midline incision was made to expose the skull, and the periosteum was removed. A headpost was positioned over the IC region (1.0 mm lateral, 5.0 mm posterior to bregma) and secured with dental cement. Ketoprofen (5 mg/kg)^60^ was administered subcutaneously for postoperative analgesia. Animals were monitored daily for signs of discomfort, and the incision site was inspected and gently treated with povidone-iodine. Additional ketoprofen was administered for 2–3 days. After 2–3 days of recovery, mice began habituation sessions for head fixation. During habituation and treadmill training (typically 7–10 days), animals continued to be monitored for any signs of discomfort or infection.

#### Measurement of locomotion-generated sounds

To characterize the sounds produced during locomotion, we recorded acoustic signals from a representative mouse walking on a passive treadmill. A microphone (CM16/CMPA, Avisoft) placed near (∼3 cm) the forepaw and acoustic signals were acquired at a sampling rate of 250 kHz using the UltraSoundGate 116Hb recording system (Avisoft). To measure sound intensity, recordings were referenced against a 4 kHz tone of known sound pressure level (SPL). Footstep events were detected by applying a threshold to the sound envelope (analytic signal), and the root-mean-square (RMS) sound pressure was calculated for each detected footstep and converted to dB SPL values (Figure 1C).

#### Skull vibration measurement

To assess whether locomotion causes vibrations that could activate cochlear hair cells via bone conduction, skull vibrations were recorded using a vibration sensor (BU-21771-000, Knowles, IL, USA), affixed to the exposed skull with UV-cured dental adhesive (Figure 1D). Vibration signals were acquired using an Intan headstage (RHD2132, Intan Technologies) and the Open Ephys acquisition system. The raw signal (5 Hz–16 kHz detection range) was notch filtered to remove 60 Hz noise. To extract the envelope, the signal was downsampled from 30,000 Hz to 1,000 Hz, rectified, and smoothed using a moving average filter with a 200 ms window (Figure 1E). Correlation coefficients were obtained between the vibration signal envelope (100 ms bins) and the treadmill speed (Figures 1F and 1G; *N* = 3 mice).

#### Cochlear histology

Following transcardial perfusion with 4% paraformaldehyde, cochleae were extracted and post-fixed overnight at 4°C. Samples were decalcified in 125 mM EDTA for 3–5 days at 4°C, and the cochlea was separated into basal, middle, and apical turns. Tissue was blocked for 2 h at room temperature in 2% bovine serum albumin and 4% goat serum. Samples were then incubated overnight at 4°C with primary antibodies: anti-Tuj1 (1:2000, mouse) for auditory nerve fibers and anti-Myosin VIIa (1:1000, rabbit) for hair cells. For visualization, Alexa Fluor-conjugated secondary antibodies (1:1000) were used. In a subset of animals, phalloidin staining was also performed to visualize hair cell stereocilia. Fluorescent images were acquired using a Leica TCS SP8 confocal microscope (40× objective) and processed using ImageJ. Cochlear histology was performed in 2 WT mice treated with DT, 2 DTR mice treated with saline, and 6 DT-injected DTR mice (Figure S1). Inner hair cells (IHCs) were quantified in the apical, middle, and basal turns of the cochlea based on confocal images. In control cochlear sections, myosin VIIa-positive IHCs were identified based on their characteristic size (approximately 9 μm in width and 16 μm in height) and elongated morphology along the radial axis. In cochlear sections from DT-injected DTR mice, myosin VIIa immunoreactivity was largely absent in the IHC layer, which was identified based on Tuj1-stained auditory afferent fibers or phalloidin-stained pillar cells (4 of 6 DTR mice). Occasional stained cells exhibited short, abnormal shapes and never showed phalloidin stained stereocilia; these cells were nevertheless included in the quantification if their height along the radial axis exceeded 10 μm. Although the outer hair cell (OHC) layer was not consistently preserved across all whole mount preparations and therefore was not quantified, the OHC layer also largely lacked myosin VIIa staining.

#### IC neural recordings

Mice were habituated to the treadmill setup for at least 7 days prior to neural recordings. Extracellular recordings were conducted in awake, head-fixed mice that could freely walk on a passive treadmill.^61^ For the craniotomy, anesthesia was induced with 3% isoflurane in an induction chamber and maintained at 1.5% through a nose cone with room air (0.5–1 L/min). Depth of anesthesia was monitored by toe pinch, and breathing was monitored throughout the surgery. Body temperature was maintained at 37°C with a heating pad. Craniotomy (∼3 mm diameter) was performed over the IC (centered at 1.0 mm lateral, 5.0 mm posterior to bregma), and the opening was sealed with a silicone elastomer (Kwik-Cast). Ketoprofen (5 mg/kg) was administered immediately after craniotomy for post-operative analgesia. Mice were allowed to recover until they were alert, responsive, and mobile before neural recordings began. We required a minimum recovery time of 1 h, although the actual recovery period typically lasted 2–4 h. The headpost was secured to a frame, and a tungsten electrode array (∼10 MΩ, 6 electrodes with ∼200 μm spacing, FHC) was inserted at 100–200 μm posterior to transverse sinus and 0.4–2.0 mm lateral to the midline to depths up to 1800 μm. Neural signals were recorded using a 16-channel headstage (RHD2132, Intan Technologies) and the Open Ephys acquisition system at 30 kHz, bandpass filtered between 600 and 6000 Hz. Locomotion was monitored using an optical sensor (SG23FH, Scitech Korea). Throughout the recording session, mice were monitored continuously via camera and direct inspection, and experiments were terminated if any signs of discomfort appeared such as reduced walking frequency.

#### Data analysis

To confirm deafness, white noise bursts (2–64 kHz, 10–90 dB SPL in 10 dB steps, 50 ms duration) were presented during multi-electrode mapping of the IC. In each mouse, neural activity was sampled at 4 depths (400, 800, 1200, 1600 μm) and 6 mediolateral positions (200 μm spacing) at each depth. Multi-unit spikes exceeding 3 × SD of baseline activity were extracted, and mean firing rates during stimulus presentation were compared to baseline rates (Figures S2). For each mouse, sound-evoked firing rates across sound intensities were first averaged across individual recording sites to generate a single rate-level curve (thick black lines in Figure 2B). These per-mouse rate-level curves were then averaged across animals for group comparison shown in Figure 2C.

For IC recordings during locomotion, spike sorting was performed offline using Offline Sorter v4 (Plexon). From bandpass filtered (600–6,000 Hz) recordings, spikes were detected using an amplitude threshold, typically set at 2–3 times the standard deviation of baseline noise. Spike waveforms were extracted and clustered using principal component analysis (PCA). Cluster separability in principle component space were assessed using multivariate ANOVA (*p* < 0.05), and only units with a refractory period violation rate below 0.5% (inter-spike interval <0.7 ms) were included.

Spontaneous activity was analyzed from ≥200 s of recordings. Walking speed was calculated by smoothing the treadmill signal using a 200 ms Hanning filter. Walking periods were defined as periods with speed greater than 2 cm/s, ignoring breaks shorter than 200 ms. Neural recordings were divided into 1-s segments and categorized as: stationary (no overlap with walking periods), walking (fully overlapping), or partial (partial overlap). Only stationary and walking segments were included in further analysis. Neurons were deemed significantly modulated if their firing rates differed significantly between walking and stationary segments (*p* < 0.01; Student’s *t* test), and only those with at least 4 walking segments were analyzed.

Modulation latency was determined by aligning neural activity to locomotion onset. To increase accuracy, locomotion onset was identified using the treadmill sensor signal (rather than speed), defined as a >5% change following ≥0.5 s of stationary period. For each movement onset, we computed a smoothed firing rate trace (100 ms window) and then averaged these traces across all onsets to obtain a time-dependent mean firing rate profile for each unit. Baseline mean and variance were estimated from 500 time points (0.5 s period) before movement onset (−1.5 s to −1.0 s). Modulation latency was defined as the first time point at which the mean firing rate exceeded (for increased neurons) or fell below (for decreased neurons) the baseline mean by more than 1 SD. For increased neurons, when the mean firing rate of the baseline period (1.5–1 s before onset) was 0 Hz or close to 0 Hz, a default value of 0.5 Hz was used. Only neurons with ≥10 qualified onset events preceded by a clear stationary period were included in this analysis.

For analysis of IC modulation across locomotion bouts, modulation direction and latency were analyzed around individual locomotion onsets in a small subset of neurons with sufficiently high firing rates to allow bout-wise analysis (*n* = 11; Figure 4). Smoothed firing rates were examined within a ±2 s window centered on each locomotion onset. For each bout, if the mean firing rate during a 0.5-s window (0 s to +0.5 s) exceeded the baseline mean by more than 1 SD (baseline defined as −1.5 s to −1.0 s), the bout was classified as an ‘increase’. If the firing rate decreased by more than 1 SD relative to the baseline mean, it was classified as a ‘decrease’. If the 1 SD threshold fell below zero, a 20% decrease from the mean was used. Bouts that did not meet either criterion were labeled as ‘not statistically significant changes’. For increased bouts, modulation latency was defined as the time point at which the firing rate crossed 2 SD above baseline. For decreased bouts, latency was defined as the time the firing rate dropped below the mean by 1 SD or below 0.5 Hz, whichever occurred first.

#### Histology for recording locations

At the conclusion of neural recordings, electrolytic lesions were made at depths of 500 μm and 1000 μm (30 μA, 10 s per location) to mark electrode tracks. Mice were perfused transcardially with 1x phosphate-buffered saline followed by 4% paraformaldehyde. Brains were extracted, post-fixed, cryoprotected in 30% sucrose, embedded in OCT, and sectioned at 40 μm using a cryostat (Leica, CM1860). Nissl staining was performed, and the sections were imaged using a slide scanner (Pannoramic Scan). Recording sites were verified based on the locations of lesions in the Nissl images (Figure 2I). Only units with recording sites within the anatomical boundaries of the IC were included in the analysis.

#### Electromyography (EMG) recording

EMG signals were recorded from the hindlimb using PFA-coated stainless steel wire (bare diameter: 0.003″, Cat. No. 791100, A-M Systems) or tungsten wire (bare diameter: 0.005″, Cat. No. 796500, A-M Systems). Under isoflurane anesthesia, a ∼3 mm hooked wire tip was inserted into the hindlimb flexor muscle (biceps femoris) using a syringe needle, and an analgesic (ketoprofen, 5 mg/kg) was administered. EMG signals were acquired using an Intan headstage (RHD2132, Intan Technologies) and the Open Ephys system. Data were bandpass filtered (20–450 Hz), notch filtered at 60 Hz, and downsampled to 2,000 Hz. To extract the signal envelope, EMG traces were rectified and smoothed using a 100-ms moving average, and then normalized to their maximum value. EMG onset was defined as the time point at which the smoothed signal exceeded 2x SD above the mean baseline activity, with a minimum variance threshold 0.03 (3% of the maximum EMG signal), calculated over a 0.5–1 s preceding period. Neurons were selected as significantly modulated if their firing rates differed significantly between walking and stationary segments (*p* < 0.05; Student’s *t* test). To compare latency between treadmill onset and EMG onset, data distribution was tested for normality in each group, followed by a paired Student’s *t* test (*p* < 0.01).

### Quantification and statistical analysis

#### Statistical analysis

All statistical analyses were performed using MATLAB. Unless otherwise indicated, statistical significance was defined as *p* < 0.05. To determine whether the activity of an IC neuron was significantly modulated during locomotion, a threshold of *p* < 0.01 (Student’s *t* test) was used. For comparisons involving medians or group means that are not normally distributed, Mann–Whitney U-tests were used. The Shapiro-Wilk test was used to assess normality of data distributions (Ahmed BenSaïda, https://www.mathworks.com/matlabcentral/fileexchange/13964-shapiro-wilk-and-shapiro-francia-normality-tests). All statistical details were provided in the corresponding figure legends and/or result sections, including the number of mice, statistical tests applied, *p* values, and measures of central tendency and dispersion.

## References

[1] Niell C.M., Stryker M.P. (2010). Modulation of Visual Responses by Behavioral State in Mouse Visual Cortex. Neuron.

[2] Saleem A.B., Ayaz A., Jeffery K.J., Harris K.D., Carandini M. (2013). Integration of visual motion and locomotion in mouse visual cortex. Nat. Neurosci..

[3] Ayaz A., Stäuble A., Hamada M., Wulf M.A., Saleem A.B., Helmchen F. (2019). Layer-specific integration of locomotion and sensory information in mouse barrel cortex. Nat. Commun..

[4] Schneider D.M., Mooney R. (2018). How Movement Modulates Hearing. Annu. Rev. Neurosci..

[5] Schneider D.M., Nelson A., Mooney R. (2014). A synaptic and circuit basis for corollary discharge in the auditory cortex. Nature.

[6] Zhou M., Liang F., Xiong X.R., Li L., Li H., Xiao Z., Tao H.W., Zhang L.I. (2014). Scaling down of balanced excitation and inhibition by active behavioral states in auditory cortex. Nat. Neurosci..

[7] McGinley M.J., Vinck M., Reimer J., Batista-Brito R., Zagha E., Cadwell C.R., Tolias A.S., Cardin J.A., McCormick D.A. (2015). Waking State: Rapid Variations Modulate Neural and Behavioral Responses. Neuron.

[8] Bigelow J., Morrill R.J., Dekloe J., Hasenstaub A.R. (2019). Movement and VIP interneuron activation differentially modulate encoding in mouse auditory cortex. eNeuro.

[9] Henschke J.U., Price A.T., Pakan J.M.P. (2021). Enhanced modulation of cell-type specific neuronal responses in mouse dorsal auditory field during locomotion. Cell Calcium.

[10] Khoury C.F., Fala N.G., Runyan C.A. (2023). Arousal and Locomotion Differently Modulate Activity of Somatostatin Neurons across Cortex. eNeuro.

[11] Vivaldo C.A., Lee J., Shorkey M., Keerthy A., Rothschild G. (2023). Auditory cortex ensembles jointly encode sound and locomotion speed to support sound perception during movement. PLoS Biol..

[12] Morandell K., Yin A., Rio R.T.D., Schneider D.M. (2024). Movement-Related Modulation in Mouse Auditory Cortex Is Widespread Yet Locally Diverse. J. Neurosci..

[13] Williamson R.S., Hancock K.E., Shinn-Cunningham B.G., Polley D.B. (2015). Locomotion and Task Demands Differentially Modulate Thalamic Audiovisual Processing during Active Search. Curr. Biol..

[14] Chen C., Song S. (2019). Differential cell-type dependent brain state modulations of sensory representations in the non-lemniscal mouse inferior colliculus. Commun. Biol..

[15] Yang Y., Lee J., Kim G. (2020). Integration of locomotion and auditory signals in the mouse inferior colliculus. eLife.

[16] Rummell B.P., Klee J.L., Sigurdsson T. (2016). Attenuation of responses to self-generated sounds in auditory cortical neurons. J. Neurosci..

[17] Schneider D.M., Sundararajan J., Mooney R. (2018). A cortical filter that learns to suppress the acoustic consequences of movement. Nature.

[18] Audette N.J., Zhou W., La Chioma A., Schneider D.M. (2022). Precise movement-based predictions in the mouse auditory cortex. Curr. Biol..

[19] Audette N.J., Schneider D.M. (2023). Stimulus-specific prediction error neurons in mouse auditory cortex. J. Neurosci..

[20] Malmierca M.S. (2004). The inferior colliculus: A center for convergence of ascending and descending auditory information. Neuroembryol. Aging.

[21] Winer J.A., Schreiner C.E. (2005).

[22] Chen C., Cheng M., Ito T., Song S. (2018). Neuronal organization in the inferior colliculus revisited with cell-type-dependent monosynaptic tracing. J. Neurosci..

[23] Aitkin L.M., Dickhaus H., Schult W., Zimmermann M. (1978). External Nucleus of Inferior Colliculus: Auditory and Spinal Somatosensory Afferents and Their Interactions. J. Neurophysiol..

[24] Aitkin L.M., Kenyon C.E., Philpott P. (1981). The Representation of the Auditory and Somatosensory Systems in the External Nucleus of the Cat Inferior Colliculus. J. Comp. Neurol..

[25] Morest D.K., Oliver D.L. (1984). The Neuronal Architecture of the Inferior Colliculus in the Cat: Defining the Functional Anatomy of the Auditory Midbrain. J. Comp. Neurol..

[26] Coleman J.R., Clerici W.J. (1987). Sources of Projections to Subdivisions of the Inferior Colliculus in the Rat. J. Comp. Neurol..

[27] Lesicko A.M.H., Hristova T.S., Maigler K.C., Llano D.A. (2016). Connectional modularity of top-down and bottom-up multimodal inputs to the lateral cortex of the mouse inferior colliculus. J. Neurosci..

[28] Olthof B.M.J., Rees A., Gartside S.E. (2019). Multiple nonauditory cortical regions innervate the auditory midbrain. J. Neurosci..

[29] Gartside S.E., Olthof B.M., Rees A. (2024). Motor, somatosensory, and executive cortical areas elicit monosynaptic and polysynaptic neuronal activity in the auditory midbrain. Hear. Res..

[30] Huey E.L., Turecek J., Delisle M.M., Mazor O., Romero G.E., Dua M., Sarafis Z.K., Hobble A., Booth K.T., Goodrich L.V. (2025). The auditory midbrain mediates tactile vibration sensing. Cell.

[31] Solyga M., Keller G.B. (2025). Multimodal mismatch responses in mouse auditory cortex. eLife.

[32] Kern A.C., Ellermeier W. (2020). Audio in VR: Effects of a Soundscape and Movement-Triggered Step Sounds on Presence. Front. Robot. AI.

[33] Cornwell T., Woodward J., Wu M., Jackson B., Souza P., Siegel J., Dhar S., Gordon K.E. (2020). Walking With Ears: Altered Auditory Feedback Impacts Gait Step Length in Older Adults. Front. Sports Act. Living.

[34] Puria S., Rosowski J.J. (2012). Békésy’s contributions to our present understanding of sound conduction to the inner ear. Hear. Res..

[35] Tong L., Strong M.K., Kaur T., Juiz J.M., Oesterle E.C., Hume C., Warchol M.E., Palmiter R.D., Rubel E.W. (2015). Selective deletion of cochlear hair cells causes rapid age-dependent changes in spiral ganglion and cochlear nucleus neurons. J. Neurosci..

[36] Fukushima M., Margoliash D. (2015). The effects of delayed auditory feedback revealed by bone conduction microphone in adult zebra finches. Sci. Rep..

[37] Fettiplace R. (2017). Hair cell transduction, tuning, and synaptic transmission in the mammalian cochlea. Compr. Physiol..

[38] Malmierca M.S., Blackstad T.W., Osen K.K. (2011). Computer-assisted 3-D reconstructions of Golgi-impregnated neurons in the cortical regions of the inferior colliculus of rat. Hear. Res..

[39] Akay T., Tourtellotte W.G., Arber S., Jessell T.M. (2014). Degradation of mouse locomotor pattern in the absence of proprioceptive sensory feedback. Proc. Natl. Acad. Sci. USA.

[40] Turecek J., Ginty D.D. (2024). Coding of self and environment by Pacinian neurons in freely moving animals. Neuron.

[41] Clayton K.K., Williamson R.S., Hancock K.E., Tasaka G.-I., Mizrahi A., Hackett T.A., Polley D.B. (2021). Auditory Corticothalamic Neurons Are Recruited by Motor Preparatory Inputs. Curr. Biol..

[42] Salvi R.J., Wang J., Ding D. (2000). Auditory plasticity and hyperactivity following cochlear damage. Hear. Res..

[43] Schormans A.L., Scott K.E., Vo A.M.Q., Tyker A., Typlt M., Stolzberg D., Allman B.L. (2016). Audiovisual temporal processing and synchrony perception in the rat. Front. Behav. Neurosci..

[44] Allman B.L., Keniston L.P., Meredith M.A. (2009). Adult deafness induces somatosensory conversion of ferret auditory cortex. Proc. Natl. Acad. Sci. USA.

[45] Harmon T.C., Madlon-Kay S., Pearson J., Mooney R. (2024). Vocalization modulates the mouse auditory cortex even in the absence of hearing. Cell Rep..

[46] Lohse M., Dahmen J.C., Bajo V.M., King A.J. (2021). Subcortical circuits mediate communication between primary sensory cortical areas in mice. Nat. Commun..

[47] Lohse M., Zimmer-Harwood P., Dahmen J.C., King A.J. (2022). Integration of somatosensory and motor-related information in the auditory system. Front. Neurosci..

[48] Lee A.M., Hoy J.L., Bonci A., Wilbrecht L., Stryker M.P., Niell C.M. (2014). Identification of a brainstem circuit regulating visual cortical state in parallel with locomotion. Neuron.

[49] Caggiano V., Leiras R., Goñi-Erro H., Masini D., Bellardita C., Bouvier J., Caldeira V., Fisone G., Kiehn O. (2018). Midbrain circuits that set locomotor speed and gait selection. Nature.

[50] Reimer J., McGinley M.J., Liu Y., Rodenkirch C., Wang Q., McCormick D.A., Tolias A.S. (2016). Pupil fluctuations track rapid changes in adrenergic and cholinergic activity in cortex. Nat. Commun..

[51] Xiao C., Cho J.R., Zhou C., Treweek J.B., Chan K., McKinney S.L., Yang B., Gradinaru V. (2016). Cholinergic Mesopontine Signals Govern Locomotion and Reward through Dissociable Midbrain Pathways. Neuron.

[52] Erisken S., Vaiceliunaite A., Jurjut O., Fiorini M., Katzner S., Busse L. (2014). Effects of locomotion extend throughout the mouse early visual system. Curr. Biol..

[53] Nelson A., Schneider D.M., Takatoh J., Sakurai K., Wang F., Mooney R. (2013). A circuit for motor cortical modulation of auditory cortical activity. J. Neurosci..

[54] Singla S., Dempsey C., Warren R., Enikolopov A.G., Sawtell N.B. (2017). A cerebellum-like circuit in the auditory system cancels responses to self-generated sounds. Nat. Neurosci..

[55] Eliades S.J., Wang X. (2008). Neural substrates of vocalization feedback monitoring in primate auditory cortex. Nature.

[56] Gruters K.G., Groh J.M. (2012). Sounds and beyond: Multisensory and other non-auditory signals in the inferior colliculus. Front. Neural Circuits.

[57] Lee T.-Y., Weissenberger Y., King A.J., Dahmen J.C. (2024). Midbrain encodes sound detection behavior without auditory cortex. eLife.

[58] Ford A.N., Czarny J.E., Rogalla M.M., Quass G.L., Apostolides P.F. (2024). Auditory Corticofugal Neurons Transmit Auditory and Non-auditory Information During Behavior. J. Neurosci..

[59] Du X., Xu H., Song P., Zhai Y., Ye H., Bao X., Huang Q., Tanigawa H., Tu Z., Chen P. (2025). The multifaceted role of the inferior colliculus in sensory prediction, reward processing, and decision-making. eLife.

[60] Huss M.K., Pacharinsak C. (2022). A Review of Long-acting Parenteral Analgesics for Mice and Rats. JAALAS.

[61] Yang Y., Kim G. (2020). Headpost Surgery for in vivo Electrophysiological Recording in the Mouse Inferior Colliculus during Locomotion. Bio. Protoc..

