# Supplementary material for: Locomotion-induced neural activity independent of auditory feedback in the mouse inferior colliculus
Source: iScience. 2026 Feb 17;29(3):115057. doi: 10.1016/j.isci.2026.115057 (PMC12964220; doi:10.1016/j.isci.2026.115057)
Supplement: Document S1. Figures S1–S3 [file mmc1.pdf]

iScience, Volume *29*

Supplemental information

Locomotion-induced neural activity independent of auditory feedback in the mouse inferior colliculus

Jisoo Han, Haiyan Jiang, Young Rae Ji, and Gunsoo Kim

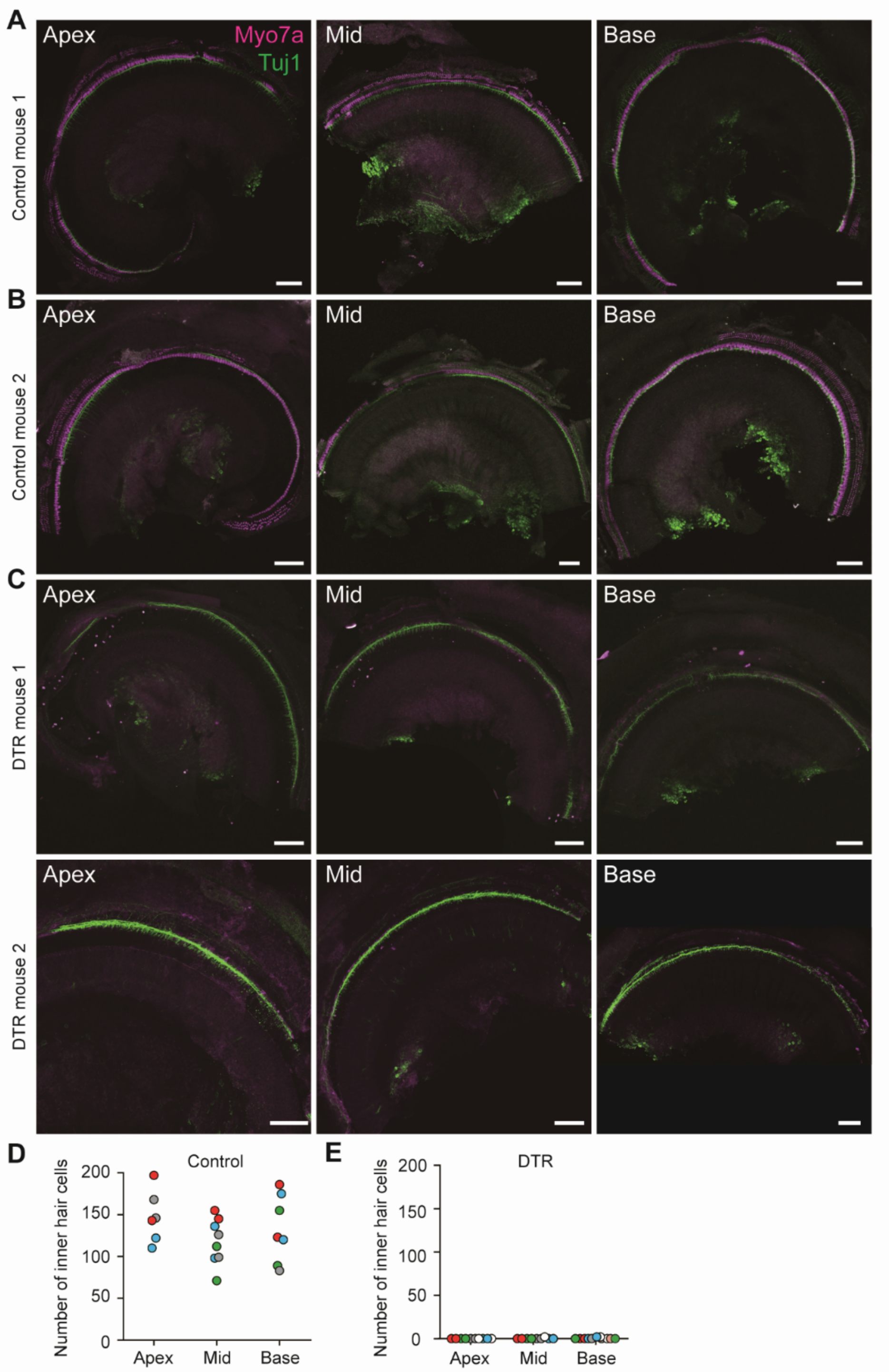

Figure S1. Histological verification of deafness in Pou4f3$^{+/DTR}$ mice following diphtheria toxin (DT) injection, related to Figure 2. (**A**) Cochlear whole mount images from a WT mouse injected with diphtheria toxin (DT), showing apical, middle, and basal turns. (**B**) Cochlear images from a DTR mice injected with saline. (**C**) Cochlear images from 2 DTR mice injected with DT. Hair cells are labeled with myosin VIIa (magenta) and auditory nerve fibers are labeled with Tuj1 (green). Scale bar, 100 µm. (**D**) Quantification of inner hair cells (IHCs) in control mice (N = 4 mice). Each color represents an individual mouse, and two dots of the same color represent sections from both ears. Not all cochlear sections were successfully retained during processing. Control groups included 2 WT mice injected with DT (blue, gray) and 2 DTR mice injected with saline (red, green). Mean IHC counts per section were 147.7 (apex), 117.8 (middle), and 133.0 (base). (**E**) Quantification of IHCs in DT-injected DTR mice (N = 6 mice). Mean IHC counts per section were 0 (apex), 0.2 (middle), and 0.4 (base).

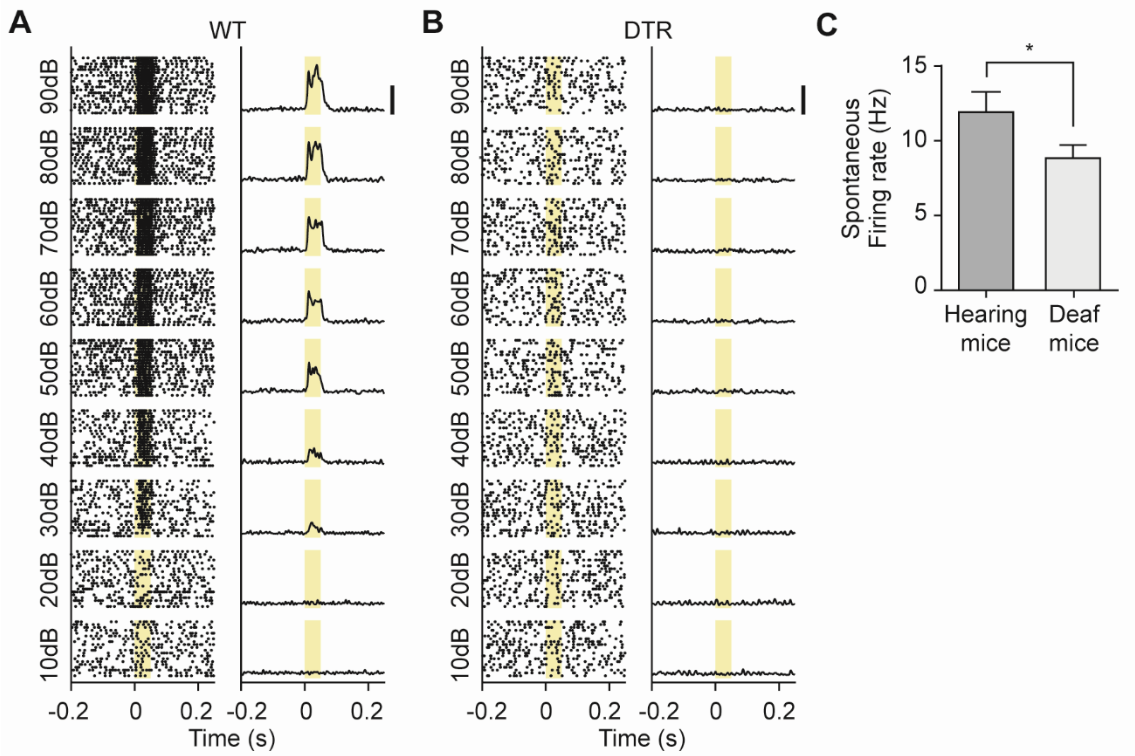

Figure S2. Functional verification of deafness in Pou4f3$^{+/DTR}$ mice following DT injection, related to Figure 2. (**A, B**) Representative multi-unit recordings from IC neurons in response to a broadband sound (50 ms, 10-90 dB SPL) in a WT mouse (C) and a DTR mouse following DT injection (D). Left panels: Raster plots showing spike times across repeated trials for each stimulus intensity. Right panels: Corresponding peristimulus time histograms (PSTHs) showing average firing rates over time. Yellow shades indicate the duration of the sound stimulus. The multi-unit data are from one of the recording sites shown in Figure 2B. Robust sound-evoked responses are observed in WT mice, but are absent in DTR mice. Scale bar, 500 Hz. (**C**) Average spontaneous firing rates in hearing (n = 73) and deaf mice (n = 146 neurons). Deaf mice exhibited significantly lower firing rates (hearing: 11.9 ± 1.4 Hz; deaf: 8.8 ± 0.9 Hz; mean ± SEM; * p = 0.0225, U = 4322, n1 = 73, n2 = 146, Mann-Whitney U-test).

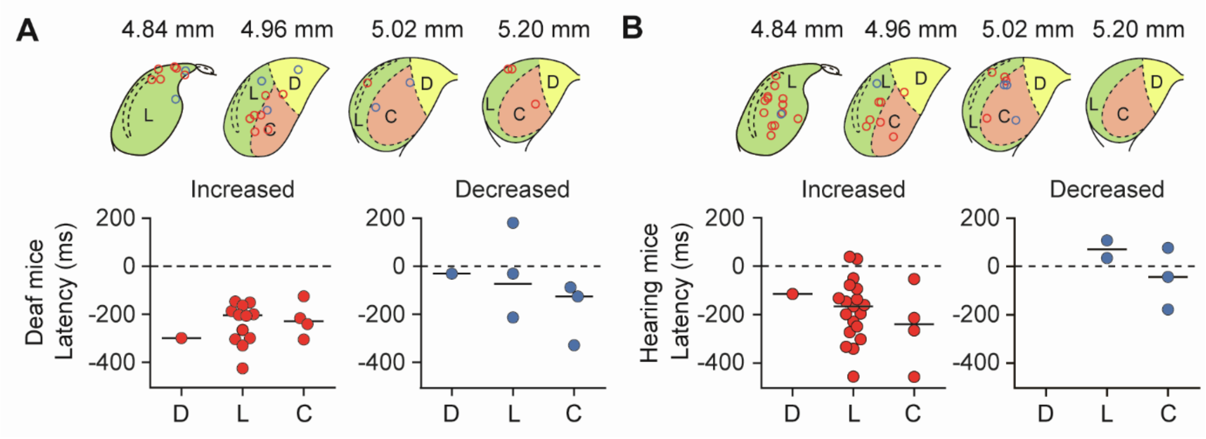

Figure S3. Spatial distribution of locomotion-induced modulation latencies in the IC of deaf and hearing mice, related to Figure 3. (**A**, **B**) Modulation latencies of IC neurons during locomotion, grouped by IC subregions in deaf (A) and hearing (B) mice. Top: Schematic diagrams of IC subdivisions at four rostro-caudal positions (D: dorsal, L: lateral, C: central). Red and blue circles represent neurons with increased and decreased firing rates during locomotion, respectively. Bottom: Latency distributions for modulated neurons within each subregion. Horizontal bars indicate median latencies. (**A**) In deaf mice, median latencies of increased neurons were −229 ms (dorsal), −205 ms (lateral), and −126 ms (central). Decreased neurons exhibited median latencies of −31 ms (dorsal), −73 ms (lateral), and −126 ms (lateral). (**B**) In hearing mice, increased neurons had median latencies of −115 ms (dorsal), −166 ms (lateral), and −240 ms (central). Decreased neurons showed latencies of: 72 ms (lateral) and −43 ms (central).
